# Enhanced psychostimulant response, but not social avoidance, depends on GluA1 AMPA receptors in VTA dopamine neurons following intermittent social defeat stress in rats

**DOI:** 10.1111/ejn.14884

**Published:** 2020-07-14

**Authors:** Megan L. Rudolph, Racheal L. Neve, Ronald P. Hammer, Ella M. Nikulina

**Affiliations:** ^1^ Department of Basic Medical Sciences University of Arizona College of Medicine Phoenix AZ USA; ^2^ Interdisciplinary Neuroscience Program Arizona State University Tempe AZ USA; ^3^ Gene Delivery Technology Core Massachusetts General Hospital Boston MA USA; ^4^ Department of Psychiatry University of Arizona College of Medicine Phoenix AZ USA; ^5^ Department of Pharmacology University of Arizona College of Medicine Tucson AZ USA

**Keywords:** amphetamine cross‐sensitization, glutamate signalling, mesolimbic, TH‐Cre rats, viral‐mediated gene transfer

## Abstract

Evidence from both human and animal studies demonstrates the importance of social stress in the development of addiction‐related behaviour. In rats, intermittent social defeat stress causes long‐lasting psychostimulant cross‐sensitization. Our recent data reveal heightened expression of AMPA receptor (AMPAR) GluA1 subunit in rat ventral tegmental area (VTA), which occurs concurrently with social stress‐induced amphetamine (AMPH) cross‐sensitization. In addition, social stress in rats induced social avoidance behaviour. The present study evaluated the effects of intermittent social defeat stress on GluA1 expression in VTA dopamine (DA) neurons, then utilized Cre‐dependent virus‐mediated gene transfer to determine the functional role of homomeric GluA1‐AMPARs in these neurons. Social defeat stress exposure induced GluA1 expression in VTA DA neurons, as demonstrated by a greater density of GluA1/tyrosine hydroxylase (TH) double‐labelling in VTA neurons in stressed rats. Additionally, functional inactivation of VTA GluA1 AMPARs in DA neurons prevented stress‐induced cross‐sensitization, or augmented locomotor response to low dose AMPH challenge (1.0 mg/kg, i.p.), but had no effect on social stress‐induced social avoidance behaviour. Furthermore, wild‐type overexpression of GluA1 in VTA DA neurons had the opposite effect; locomotor‐activating effects of AMPH were significantly augmented, even in the absence of stress. Taken together, these results suggest that stress‐induced GluA1 expression in VTA DA neurons is necessary for psychostimulant cross‐sensitization, but not for social avoidance. This differential effect suggests that different neural pathways are implicated in these behaviours. These findings could lead to novel pharmacotherapies to help prevent stress‐induced susceptibility to substance abuse.

## Graphical abstract

Functional inactivation of GluA1‐homomeric AMPA receptors in VTA dopamine neurons of TH‐Cre rats prevents social stress‐induced sensitization to amphetamine, but has no effect on stress‐induced social avoidance behavior. Overexpression of GluA1‐homomeric AMPA receptors in VTA dopamine neurons of TH‐Cre rats mimics the effects of social stress on amphetamine sensitivity, inducing augmented locomotor activity following low‐dose amphetamine challenge.

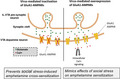

**Abbreviations**
AMPA: α‐amino‐3‐hydroxy‐5‐methyl‐4‐isoxazoleproionic acid
BDNF: brain derived neurotrophic factor
CaMKII: Ca^2+^/calmodulin dependent protein kinase II
DA: Dopamine
fr: fasciculus retroflexus
GABA: gamma aminobutyric acid
LTP: long‐term potentiation
NAc: nucleus accumbens
NMDA: N‐methyl‐D‐aspartate
TH: tyrosine hydroxylase
VTA: ventral tegmental area

## 1 INTRODUCTION

Stress is an influential factor that impacts the transition from recreational drug use to addiction and has been correlated with increased substance abuse susceptibility and relapse (Sinha, 2001, 2008, 2011). Converging evidence from human and animal studies demonstrates that repeated social stress augments the locomotor effect of psychostimulants in a phenomenon referred to as “cross‐sensitization” (Covington & Miczek, 2001; Nikulina, Covington, Ganschow, Hammer, & Miczek, 2004; Nikulina, Lacagnina, Fanous, Wang, & Hammer, 2012) and leads to social avoidance behaviour (Berton et al., 2006; Fanous, Terwilliger, Hammer, & Nikulina, 2011; Komatsu et al., 2011). These social stress‐induced behavioural effects are long‐lasting (Covington et al., 2005; Nikulina et al., 2004) and induce prolonged activation of mesocorticolimbic pathways, which are comprised of dopaminergic neurons that originate in the ventral tegmental area (VTA; Swanson, 1982).

In rodents, psychostimulant administration increases mesocorticolimbic glutamate transmission in the nucleus accumbens (NAc), striatum and VTA (Del Arco, Gonzalez‐Mora, Armas, & Mora, 1999; Reid, Hsu, & Berger, 1997; Wolf & Xue, 1998, 1999; Xue, Ng, Li, & Wolf, 1996). This glutamate input, mostly originating from cortical regions, provides excitatory control of VTA dopamine (DA) neuronal activity (Johnson & North, 1992; Sesack & Pickel, 1992; Taber & Fibiger, 1995), binding to α‐amino‐3‐hydroxy‐5‐methyl‐4‐isoxazoleproionic acid (AMPA) or N‐methyl‐D‐aspartate (NMDA) receptors. AMPA receptors (AMPARs) are ionic transmembrane receptors with four subunits, GluA1‐4, which modulate receptor trafficking and channel functions. AMPARs comprised of GluA1 homodimers (i.e., GluA2‐lacking) are Ca^2+^‐permeable and have higher single‐channel conductance, enhancing Ca^2+^‐dependent intracellular signalling (Straub & Tomita, 2012; Wolf, 2010; Wolf & Tseng, 2012). Induction of psychostimulant sensitization is blocked by AMPAR antagonist administration (Li, Vartanian, White, Xue, & Wolf, 1997; Vanderschuren & Kalivas, 2000; Zhang, Hu, White, & Wolf, 1997), and higher VTA GluA1 levels are implicated in drug‐induced sensitization (Carlezon & Nestler, 2002; Kalivas, 2009; Ping, Xi, Prasad, Wang, & Kruzich, 2008). Although evidence has implicated a critical role of GluA1 in drug‐induced sensitization, it is unknown whether GluA1 in VTA DA neurons plays a causal role in social stress‐induced sensitization to psychostimulants. Western blot data demonstrate that social stress induces VTA GluA1 expression, which occurs concomitantly with social stress‐induced AMPH cross‐sensitization (Covington, Tropea, Rajadhyaksha, Kosofsky, & Miczek, 2008; Wang et al., 2014). However, the cellular localization of induced GluA1 expression by social stress exposure is not known.

To address this question, we first evaluated the effects of intermittent social defeat stress on GluA1 expression in VTA DA neurons. To determine the role of GluA1 in VTA DA neurons in stress‐induced AMPH sensitivity and social avoidance behaviour, we used TH‐Cre rats and utilized Cre‐inducible AAV‐mediated gene transfer to bidirectionally manipulate GluA1 expression preferentially in VTA DA neurons. We performed intra‐VTA infusion of a viral construct that expresses pore‐dead GluA1 in a dominant‐negative manner to functionally inactivate GluA1 AMPARs. We also used a viral construct that expresses wild‐type (wt) GluA1 to overexpress GluA1 AMPARs in VTA DA neurons. By doing so, we were able to test the causal role of GluA1 in VTA DA neurons on social stress‐induced sensitization to psychostimulants. To reveal the behavioural specificity of VTA GluA1 signalling for the sensitized response to psychostimulants, we also studied the effect of intra‐VTA GluA1 manipulation on intermittent social stress‐induced social avoidance behaviour.

## 2 MATERIALS AND METHODS

### 2.1 Subjects

Experimental subjects were male Sprague‐Dawley homozygous tyrosine hydroxylase (TH)‐Cre rats (Sage Laboratories) weighing 200–250 g at the start of experimentation. Two breeding pairs of TH‐Cre Sprague‐Dawley rats were ordered from SAGE laboratory, where they were verified to be homozygous and to have no observed ectopic expression of Cre (Sage Laboratories). All experimental animals were bred on site at the University of Arizona College of Medicine‐Phoenix animal facility and were maintained under a reverse light/dark cycle (12 hour:12 hour, lights on at 9:00 a.m.), with ad libitum access to food (Purina Rodent Diet) and water. Three days prior to the first social stress exposure, subjects were individually housed in standard plastic cages (25 × 50 × 20 cm^3^). Male Long‐Evans rats (weighing 550–700 g) termed “residents”, were pair‐housed with female Long‐Evans rats in larger plastic cages (37 × 50 × 20 cm^3^). Residents were screened for aggressive behaviour as described previously (Nikulina et al., 2012) and were used to induce social defeat stress in the experimental “intruder” TH‐Cre rats as described below. All experimental procedures were approved by the University of Arizona Institutional Animal Care and Use Committees. All studies were conducted in accordance with the Guide for the Care and Use of Laboratory Animals (National Research Council, 2011), and efforts were made to minimize pain and suffering and reduce the number of animals used.

### 2.2 Viral vectors

Rats assigned to the control viral group received bilateral infusions of adeno‐associated viral (AAV) constructs that express green fluorescent protein (GFP) and a Cre insert (AAV5‐CMV‐HI‐eGFP‐Cre‐WPRE‐SV40; AAV‐GFP; viral titre: 1.864E + 13 vg/ml; Penn Vector Core) to identify the injection site. Rats assigned to the VTA GluA1 functional inactivation group received a Cre‐dependent AAV that expresses dominant‐negative pore‐dead GluA1 (AAV5.2‐hEF1a‐DIO‐GluA1‐Q58IE‐SV40PA; AAV‐pd‐GluA1; viral titre: 2.24E + 13 vg/ml; Gene Technology Core, Massachusetts General Hospital), containing a single point mutation (Q581E) in the pore region, which reduces synaptic AMPA currents through heteromeric interactions with endogenous AMPA subunits (Dingledine, Borges, Bowie, & Traynelis, 1999; Shi, Hayashi, Esteban, & Malinow, 2001). Rats assigned to the GluA1 overexpression group received a Cre‐dependent AAV that expresses wild‐type GluA1 (AAV5.2‐hEF1a‐DIO‐Rev‐GluA1‐wt‐SV40PA; AAV‐wt‐GluA1; viral titre: 2.13E + 13 vg/ml; Gene Technology Core, Massachusetts General Hospital). Both viral vectors manipulating GluA1 expression were previously validated in vitro and in vivo (Bachtell et al., 2008).

### 2.3 Bilateral intracranial viral infusions

After random group assignment, rats were anesthetized using isoflurane and positioned in a stereotaxic frame (Leica Angle Two). The respective viral construct was bilaterally infused (1.0 µl per side) into the VTA (AP −5.0 to −5.3, ML ± 2.1, DV −8.2, tilt 10°; Paxinos & Watson, 2007) at a constant flow rate of 0.1 µl/min (Hamilton; Model 7105 KH; 24‐gauge tip). After infusion, the needle remained at the infusion site for 5 min to prevent retrace of the virus. Animals that were assigned to non‐viral control groups received sham surgeries, in which they were anesthetized with isoflurane and were positioned in a stereotaxic frame, but no surgery was performed. Rats recovered in their cages for 2–3 weeks to allow for optimal viral expression before the start of social stress or handling procedures.

Viral infusion site localization was performed using fluorescent immunohistochemistry (described below) after each experiment to ensure accurate viral expression in medial/anterior portions of the VTA, which are known to have a high density of DA neurons (Morales & Margolis, 2017).

### 2.4 Experimental design

**Figure 1:**
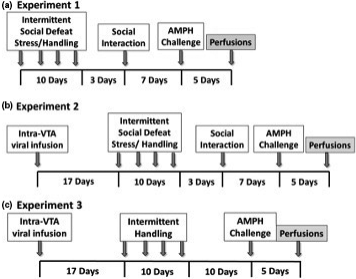
Schematic timelines of experimental design. (a) Experiment 1: Characterization of VTA neurons in which GluA1 induction occurs after intermittent social defeat stress. (b) Experiment 2: Effect of virus‐mediated inactivation of GluA1 in VTA dopamine neurons on behavioural sensitization to AMPH. Experiment 3: Effect of viral overexpression of GluA1 in VTA dopamine neurons on behavioural sensitization to AMPH in handled animals

We conducted three separate experiments that were designed to elucidate the mechanisms underlying the involvement of GluA1 localized to VTA DA neurons in stress‐induced AMPH sensitization and social interaction (Figure 1).

#### 2.4.1 Experiment 1: Characterization of VTA neurons in which GluA1 induction occurs after social stress and AMPH administration

Rats were exposed to social stress (*n* = 13) or handling (*n* = 12), then received d‐AMPH sulphate (1.0 mg/kg, i.p.; Sigma‐Aldrich; in 0.9% saline) 10 days after the last stress or handling procedure, known to produce long‐term behavioural effects of intermittent social defeat stress in rats (Covington & Miczek, 2001; Miczek, Nikulina, Shimamoto, & Covington, 2011; Nikulina et al., 2004). Brain tissue was collected and processed 5 days after the AMPH challenge to ensure that the drug was completely removed from the rats’ system in order to measure the effects of stress rather than AMPH challenge. GluA1/TH colocalization was assessed in the VTA (Figure 1a).

#### 2.4.2 Experiment 2: Effect of virus‐mediated inactivation of GluA1 in VTA DA neurons on behavioural sensitization to AMPH and social interaction

Rats were randomly assigned to one of four groups based on two experimental factors: virus (AAV‐pd‐GluA1 vs. sham infusion) or behavioural treatment (handling vs. social stress). The groups were as follows: sham‐handled (*n *= 17), sham‐stressed (*n *= 15), AAV‐GluA1‐pd‐handled (*n* = 15) and AAV‐pd‐GluA1‐stressed (*n* = 15). These rats were subjected to a social interaction test 3 days after termination of social stress and handling procedures, then were given AMPH challenge 10 days after the last stress or handling procedure. Animals were euthanized 5 days after AMPH challenge, at which time brains were collected for processing. Locomotor activity was analysed, and immunohistochemistry was performed to examine GluA1/TH double‐labelling in the VTA (Figure 1b).

#### 2.4.3 Experiment 3: Effect of viral overexpression of GluA1 in VTA DA neurons on behavioural sensitization to AMPH in handled animals

Rats were randomly assigned to groups based on viral vector (AAV‐wt‐GluA1, AAV‐GFP or sham), and all animals received handling. The experimental groups were as follows: sham (*n *= 8), AAV‐GFP (*n* = 5) and AAV‐wt‐GluA1 (*n* = 14). These subjects were challenged with AMPH 10 days after the last handling procedure. Rats were euthanized 5 days later, and brains were collected for immunohistochemical processing. Locomotor activity was analysed, and GluA1/TH double‐labelling in the VTA was assessed (Figure 1c).

### 2.5 Intermittent social defeat stress and handling procedure

Social stress was induced by a brief confrontation between an aggressive resident rat and an experimental intruder rat, as previously described (Nikulina et al., 2012). This defeat procedure was performed in a sound‐attenuated room to prevent ultrasonic stimuli from affecting unstressed subjects. After removing the female from the resident’s home cage, an experimental intruder rat was placed inside the resident’s home cage for 5 min under a small metal protective cage (15 × 25 × 15 cm^3^). The protective cage was then removed, allowing the resident to attack the experimental rat until it displayed a submissive supine posture for at least 6 s, or for 5 min, whichever came first. The experimental rat was then placed back into the protective cage for an additional 20 min before returning to its home cage. The social stress procedure was performed every third day for 10 days, producing intermittent exposure to social defeat stress. On the same days as social stress, rats in the control groups were handled for 2–3 min and returned to their home cages.

### 2.6 Social interaction

The social approach/avoidance test was conducted in a three‐chamber container (58 × 38 × 41 cm^3^), with lightweight metal cages on either side (Figure 5a). Experimental rats were allowed to habituate to the chamber for 5 min and then were reintroduced to the “neutral” zone when a novel Long‐Evans rat was placed under the containment cage on one side of the three‐chamber container. The behaviour of the rat was recorded using Ethovision video tracking software (Noldus), which separated the chamber into three zones: the interaction zone (the side that contains the novel rat), the neutral zone (the middle) and the avoidance zone (the side that contains an empty containment cage). The number of respective entries into the avoidance and interaction zones were recorded, as well as the total distance travelled (cm).

### 2.7 AMPH challenge

To test for social stress‐induced cross‐sensitization, a low dose of AMPH was administered as previously described (Nikulina et al., 2012). Rats were injected with vehicle (0.9% sterile saline; 1.0 ml/kg, i.p. daily) on several days prior to the AMPH challenge to acclimate them to i.p. injections. On the day of the AMPH challenge, rats were moved into the procedure room, where locomotor activity measured as total distance travelled (in cm) was recorded in their home cage during sequential 10 min bins using Ethovision. Baseline locomotor activity was recorded for 30 min, after which a 0.9% saline injection (1.0 ml/kg, i.p.) was administered and locomotor activity was recorded for 40 min. Finally, rats received an injection of D‐AMPH sulphate (1.0 ml/kg in saline vehicle, i.p.), and locomotor activity was recorded for an additional 60 min.

### 2.8 Tissue harvesting for immunohistochemistry

As previously described (Fanous et al., 2011), rats were deeply anesthetized with sodium pentobarbital (100 mg/kg, i.p.; Euthasol, Virbac Co.) and perfused transcardially with 4% paraformaldehyde. Brains were extracted, post‐fixed for 90 min at 4°C and placed into graded sucrose solutions. Frozen brain tissue was sectioned on a cryostat (20 µm) and serial VTA sections (AP = −5.0 to −5.6, Paxinos & Watson, 2007) were mounted onto slides. Adjacent VTA slides from each brain were processed for either GluA1/ TH double‐labelling or fluorescent localization of GFP expression.

### 2.9 Immunohistochemistry

To determine the cellular localization of GluA1 expression in VTA DA neurons, we performed fluorescent double‐labelling of GluA1 and TH. Sections were first washed in 0.05 M potassium phosphate buffered‐saline (KPBS), then blocked for 1 hr in 10% normal goat serum and 0.4% Triton X‐100 in 0.05 M KPBS. Sections were then incubated simultaneously with rabbit anti‐GluA1 (ABN241, 1:500 dilution; Millipore), which recognizes both active and inactive forms of GluA1, and mouse anti‐TH (SC‐7837, 1:500 dilution; Santa Cruz Biotechnology) at 4°C for 48 hr. Slides were then incubated with biotinylated goat anti‐rabbit IgG (1:1,000 dilution, Vector Laboratories) for 1 hr and then Alexa Fluor 488 Anti‐Rabbit conjugated streptavidin and Alexa Fluor 647 Goat Anti‐Mouse (1:1,000 dilution; Invitrogen) were applied for 2.5 hr. After washing with 0.05 M KPBS, coverslips were applied with ProLong Diamond Antifade Mountant (Invitrogen).

### 2.10 Modified stereological quantification

Tissue sections were imaged using a Zeiss Axioscope with a 20× objective lens and were digitalized using a colour digital camera. The number of GluA1/TH double‐labelled neurons was quantified using ImageJ software (NIH), and the analysis was conducted using a modified stereology counting procedure as described previously (Fanous et al., 2011; Nikulina et al., 2012). A grid of 30 squares (0.0075 mm^2^) was superimposed on 2–3 adjacent VTA sections bilaterally from each subject. Double‐labelled neurons were counted in 15 grid random squares such that labelled cells intersecting the bottom or right lines of each square were included, while cells intersecting the top or left lines of the square were excluded from the analysis. Double‐labelled cell density (in mm^2^) was calculated by averaging the bilateral counts across sections, then dividing the total number of counted cells by the total area that was assessed (0.111 mm^2^). Furthermore, total TH neuronal population was approximated by averaging the bilateral counts of TH‐labelled neurons across all sections, then dividing the total number of counted cells by the total area assessed (0.111 mm^2^). To account for any potential differences in tissue quality, the percentage of GluA1/TH double‐labelled neurons out of the entire TH neuronal population was calculated by dividing the number of GluA1/TH double‐labelled cells by the corresponding total number of TH‐labelled cells and then multiplying by 100 to obtain the percentage.

### 2.11 Statistical analyses

Across all experiments, the results are expressed as mean ± standard error (*SEM*) and a *p* value ≤.05 was considered significant. GraphPad Prism version 7 (GraphPad Prism) was used to perform all statistical analyses, and Tukey’s HSD was the preferred post hoc test across all experiments, except in the AMPH challenge, where we utilized the more conservative Fisher’s LSD test. In experiment 1, a two‐way ANOVA was used to analyse locomotor activity during the AMPH challenge, and a paired two‐tailed *t* test was used to analyse double‐labelled cell counts in stressed and handled rats. To calculate the average distance travelled during each portion of the AMPH challenge (acclimation, saline, AMPH administration), the average distance travelled between three consecutive time points was taken. After AMPH administration, the average distance travelled between the three peak time points was taken (20–40 min after AMPH administration). A two‐way ANOVA was then performed to compare the average distance travelled between experimental groups (stressed and handled) and across time points (acclimation, saline, AMPH). In experiment 2, a three‐way ANOVA was used to analyse locomotor activity (between‐subjects factors: viral vector (AAV‐pd‐GluA1 or sham), behavioural treatment (handling or stress) and experimental time point (acclimation, saline and AMPH), while a two‐way ANOVA was used to analyse immunohistochemical labelling density. Average distance travelled across stages of the AMPH challenge were calculated as described in experiment 1. In experiment 3, two‐way ANOVA was used to analyse locomotor activity and immunohistochemical labelling density (between‐subjects factors: viral vector (AAV‐wt‐GluA1, AAV‐GFP or sham). Average distance travelled across stages of the AMPH challenge were calculated as described in experiment 1. In all experiments, data were excluded in the case of video tracking errors (*n* = 3), incorrect viral infusion sites (*n* = 5), or loss of data due to damaged tissue sections (*n* = 6).

## 3 RESULTS

### 3.1 Experiment 1

#### 3.1.1 Intermittent social defeat stress induces sensitization to AMPH

**Figure 2:**
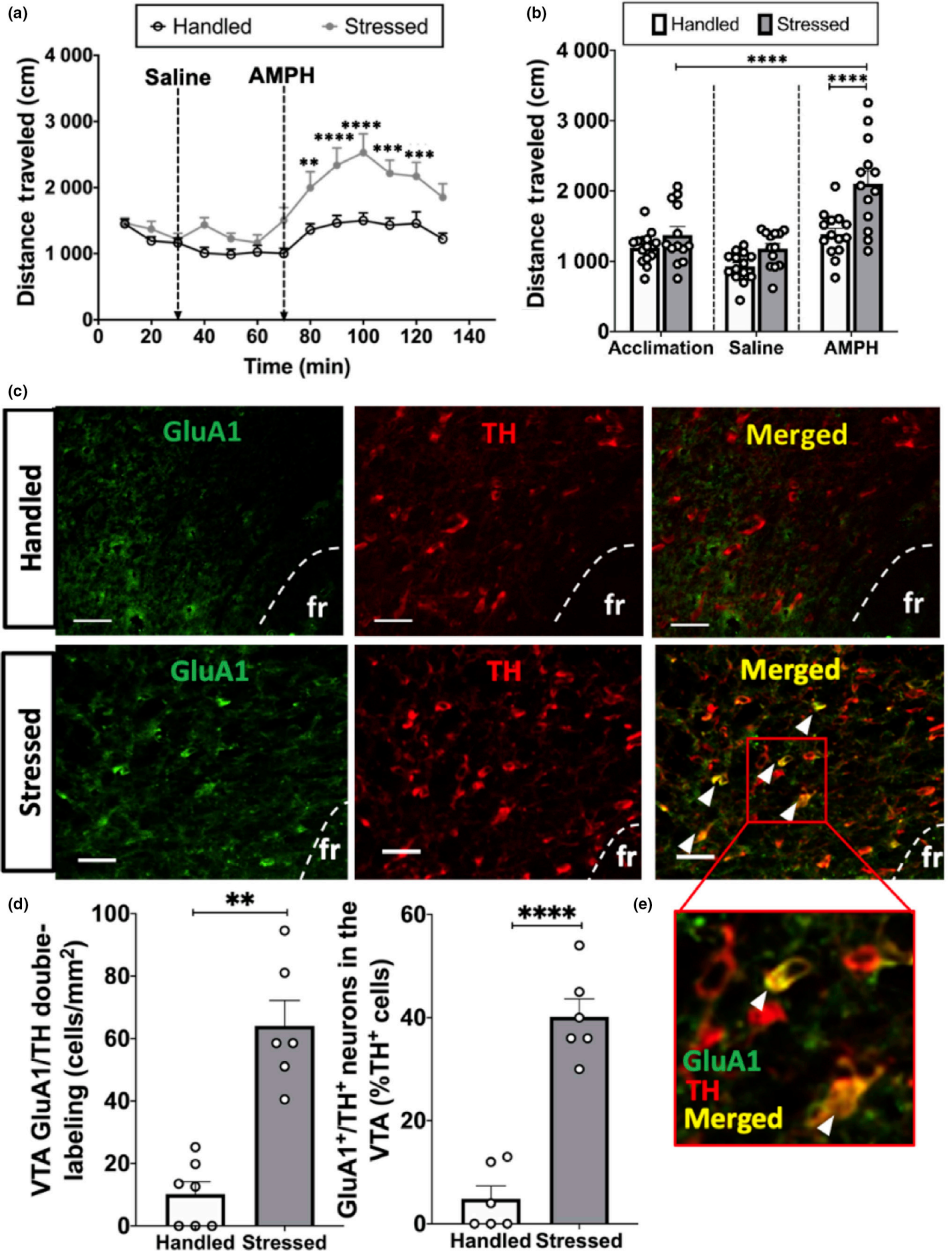
Intermittent social defeat stress induces higher GluA1 expression in VTA dopamine neurons, which occurs concomitantly with stress‐induced AMPH cross‐sensitization. (a) Locomotor activity (total distance travelled in cm) over time before and after saline, and following AMPH administration (1.0 mg/kg, i.p.). Injection times are denoted by vertical arrows. Stressed animals had significantly higher locomotor activity compared to handled animals (***p < .0005, **p < .005). (b) There was no difference in locomotor activity between baseline (acclimation) and after saline injections, but stressed rats travelled significantly more than handled rats in response to the AMPH challenge, which was significantly higher than distance travelled during baseline (****p < .0001). (c) Representative images of fluorescent GluA1 labelling (left), TH labelling (centre), and GluA1/TH double‐labelling (right) in handled (top) and stressed (bottom) animals; bar = 50 µm; arrow: GluA1/TH double‐labelled cell. (d) GluA1/TH double‐labelling in VTA is significantly higher in animals subjected to intermittent social defeat stress compared to handled animals (**p < .005). (e) Zoomed in fluorescent image of GluA1/TH double‐labelling (white arrow: GluA1/TH double‐labelled cell)

Locomotor response to AMPH challenge was evaluated in naïve rats 10 days after the last exposure to intermittent social defeat stress or handling. A two‐way ANOVA revealed that rats exposed to intermittent social defeat stress exhibited significantly more locomotor activity after AMPH than did handled rats (*n* = 27, *F*_12, 325_ = 3.16, *p* = .0013), reflecting the presence of cross‐sensitization (Figure 2a). Specifically, post hoc analysis revealed that stressed rats displayed greater locomotor activity than did handled rats at 10 (*p* = .0019), 20 (*p* = <.0001), 30 (*p* < .0001), 40 (*p* = .0001), 50 (*p* = .0005) and 60 (*p* = .0021) min after AMPH challenge, but there were no differences across groups before or after saline injection (*p* > .05 at all other time points). Additionally, a two‐way ANOVA was performed to compare the average distance travelled during acclimation, following saline treatment, and after AMPH administration, in handled versus stressed rats. This two‐way ANOVA revealed significant main effects of stage of AMPH challenge (*n* = 27, *F*_2,75_ = 32.81, *p* < .0001) and stress (*F*_1,75_ = 26.5, *p* < .0001) and an interaction between the two factors (*F*_2,75_ = 8.756, *p* = .0004) (Figure 2b).

#### 3.1.2 Intermittent social defeat stress increases GluA1 expression in VTA DA neurons

To characterize the cell types in which GluA1 induction occurs after intermittent social defeat stress, double‐label fluorescent immunohistochemistry was performed in the rostral VTA (Figure 2c,d). A paired *t* test was performed to compare the mean number of GluA1‐expressing DA neurons in stressed compared to handled rats. Significantly more GluA1/TH double‐labelled cells were present in the rostral VTA in stressed rats compared to handled rats (*n* = 13; *t* test: *p* = .0021; Figure 2d).

### 3.2 Experiment 2

#### 3.2.1 Verification of GluA1‐pd overexpression in VTA DA neurons using fluorescent immunohistochemistry

**Figure 3:**
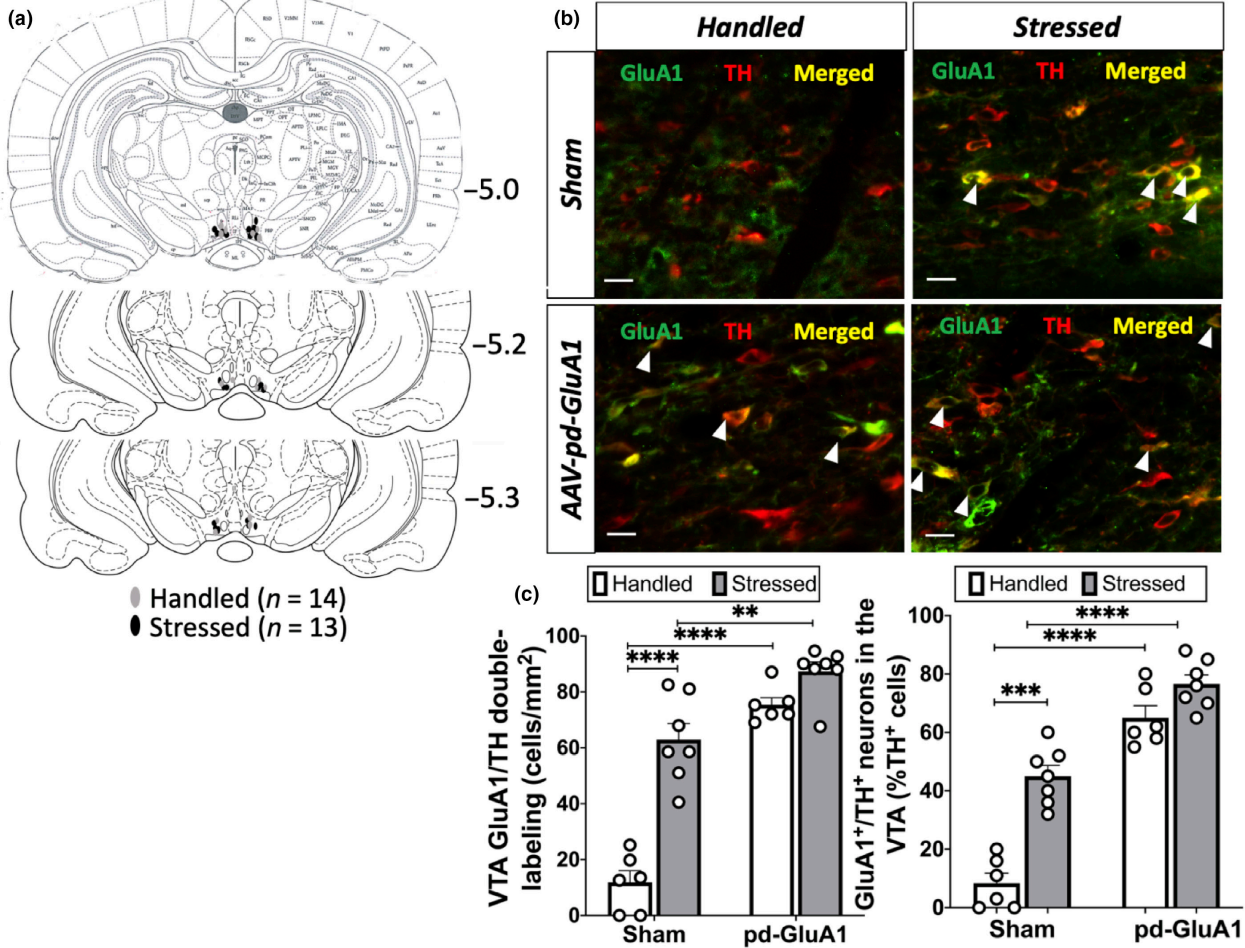
Verification of viral infusion sites: bilateral AAV‐pd‐GluA1 infusions into the VTA increases GluA1 expression in VTA DA neurons. (a) Schematic depicting all viral infusion sites between −5.0 and −5.3 mm from bregma (*n* = 14 handled rats, *n* = 13 stressed rats). (b) Because GluA1 antibodies recognize both active and inactive GluA1 AMPARs, representative fluorescent images show higher GluA1/TH double‐labelling in handled (left) and stressed (right) rats after cre‐dependent AAV‐pd‐GluA1 infusions in TH‐Cre rats (bar = 50 µm; white arrow: GluA1/TH double‐labelled cell). (c) GluA1/TH double‐labelling in VTA is significantly higher in animals subjected to intermittent social defeat stress compared to handled animals, and rats with AAV‐pd‐GluA1 infusions showed significantly more GluA1/TH double‐labelling than sham animals (**p < .005, *** *p* < .0005, ****p < .0001)

One important caveat of this experiment is that the AAV‐pd‐GluA1 and AAV‐wt‐GluA1 constructs were too large to insert a fluorescent marker such as GFP, which makes it more difficult to control for the specificity and efficiency of the viral transfections. For this reason, fluorescent immunohistochemistry was used to verify GluA1 expression in VTA DA neurons of sham rats compared to experimental rats. Fluorescent immunohistochemistry revealed that AAV‐pd‐GluA1 infusions increased GluA1 expression in VTA DA neurons (Figure 3b). A two‐way ANOVA demonstrated significant main effects of stress (*n* = 26, *F*_1,22_ = 53.53, *p* < .0001) and viral manipulation group (*F*_1,22_ = 104.2; *p* < .0001) and an interaction between the two factors (*F*_1,22_ = 20.56; *p* = .0002). Consistent with previous findings, post hoc analysis revealed that stressed rats displayed greater GluA1/TH double‐labelling than did handled rats (*p* < .0001). Furthermore, AAV‐pd‐GluA1/stressed rats exhibited significantly more GluA1 labelling in VTA DA neurons than did sham/stressed rats (*p* = .0021) and AAV‐pd‐GluA1/handled rats displayed more GluA1/TH double‐labelling than did sham/handled rats (*p* < .0001). Thus, bilateral AAV‐pd‐GluA1 infusions were accurately performed in the VTA (Figure 3a) and viral infusions increased GluA1 expression in DA neurons.

#### 3.2.2 Functional GluA1 inactivation in VTA DA neurons prevents social stress‐induced AMPH sensitization, but has no effect on social interaction behaviour

**Figure 4:**
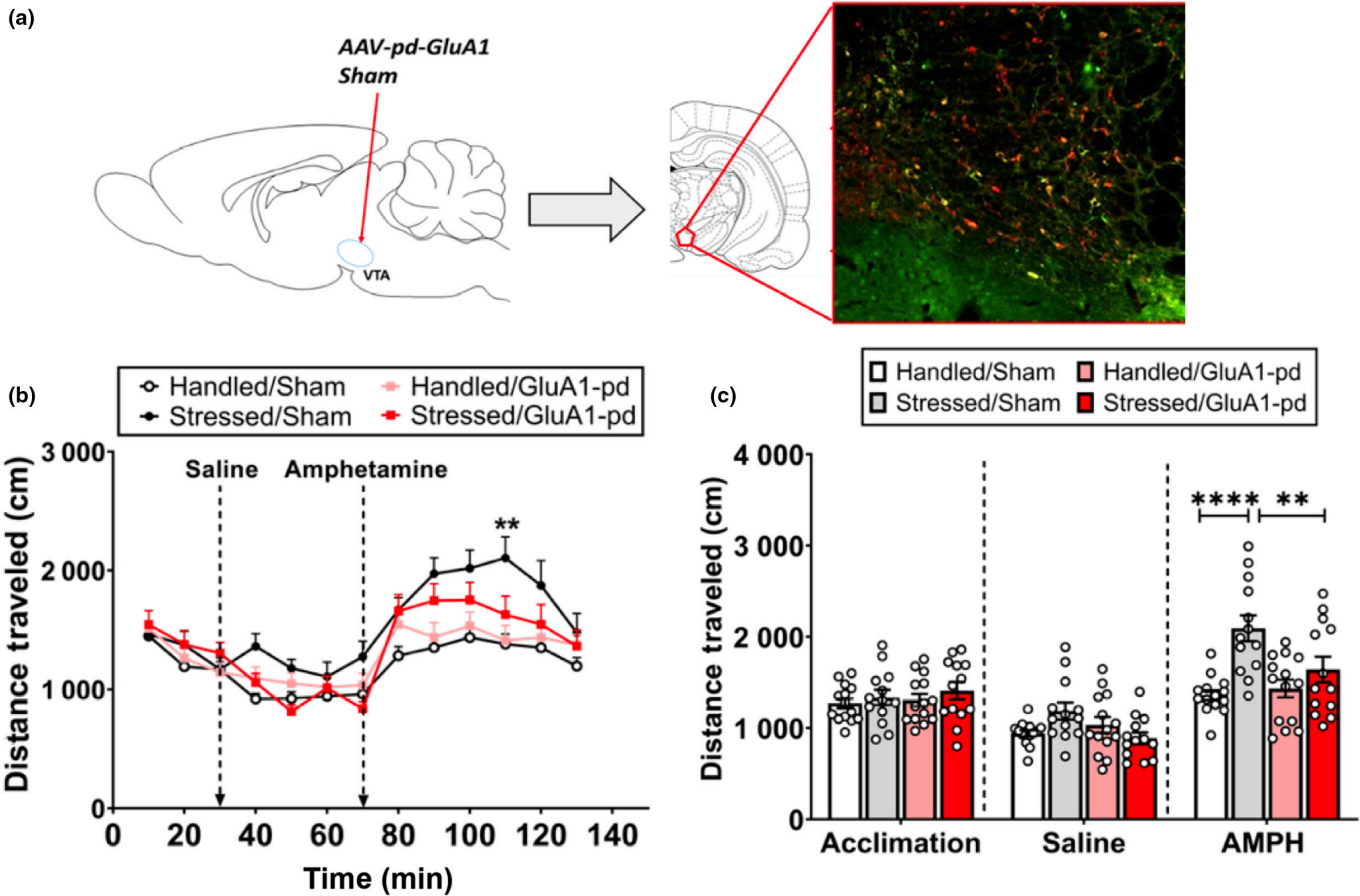
Functional inactivation of GluA1 in VTA dopamine neurons prevents stress‐induced AMPH sensitization. (a) Schematic that depicts the viral infusion site (left), and representative fluorescent GluA1/TH double‐labelling (right) in rats with viral infusions. (b) Sham‐Stressed rats travelled a significantly greater distance at 110 min (**p < .005) and 120 min (*p < .05) compared to all other groups. (c) There was no difference in average locomotor activity between groups during the acclimation period or after saline injections, but sham‐stressed rats travelled a significantly (****p < .0005; **p < .005) greater distance than handled sham rats and stressed‐GluA1‐pd rats

Three‐way ANOVA demonstrated significant main effects of stress (*n* = 62, *F*_1,650_ = 45.85, *p* < .0001) and experimental time point (*F*_12,650_ = 22.67, *p* < .0001), but no significant effect of viral construct (*F*_1,650_ = 1.952, *p* = .1628); however, there was an interaction between stress and viral construct (*F*_1,650_ = 22.58, *p* < .0001; Figure 4b)_._ Specifically, post hoc analysis revealed that AAV‐pd‐GluA1/stressed rats exhibited significantly less locomotor activity 40 min (*p* = .0024) and 50 min (*p* = .0363) after AMPH challenge than did sham/stressed rats (Figure 4a,b). Additionally, a three‐way ANOVA was performed to compare the average distance travelled during acclimation, following saline treatment, and after AMPH challenge in the different experimental groups. Three‐way ANOVA revealed a significant main effect of stress (*F*_1,150_ = 15.30, *p* = .0001) and experimental time point (*F*_2,150_ = 48.21, *p* < .0001), but no significant effect of viral construct (*F*_1,150_ = 2.362, *p* = .1265) on locomotor activity. In addition, there was an interaction between stress and viral construct on locomotor activity (*F*_1,150_ = 8.136, *p* = .0050; Figure 4b).

**Figure 5:**
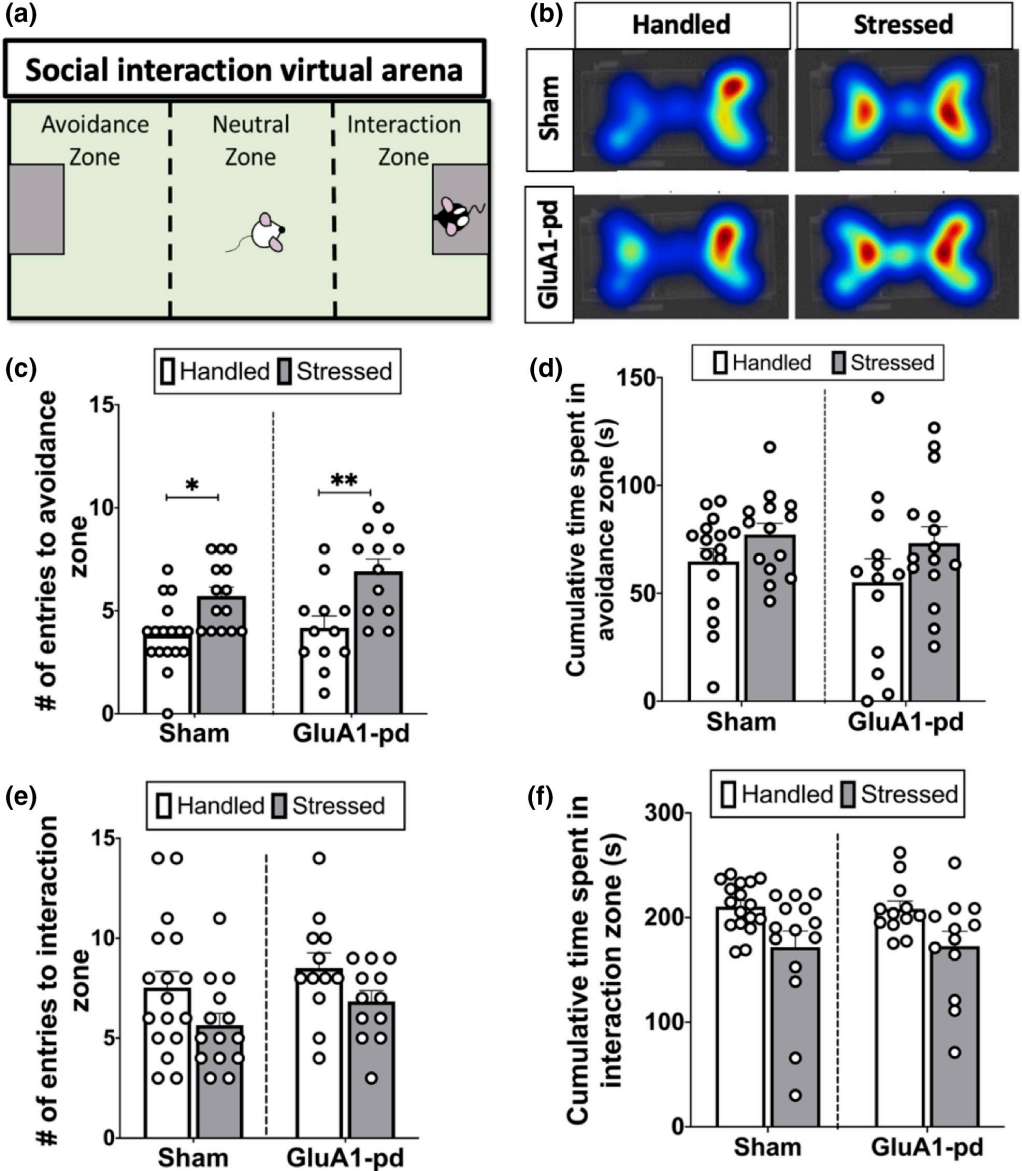
Functional GluA1 inactivation has no effect on social stress‐induced social avoidance behaviour. (a) Schematic of the social interaction test arena, showing the three‐chamber cage separated into the avoidance zone (left), neutral zone (middle), and interaction zone (right). The experimental animal was placed into the neutral zone, and then Ethovision software recorded the number of times the animal entered the avoidance zone, neutral zone, and interaction zone, and the total duration of time spent in each respective zone. (b) Representative heat maps during the social interaction test, where warmer colours indicate more time spent. (c) Stressed rats made significantly more entries into the avoidance zone than handled rats (**p < .005), but there was no difference in the number of entries into the avoidance zone between sham and AAV‐pd‐GluA1‐infused rats (*n* = 12–17 rats per group). (d) There was a trend for a higher cumulative duration of time spent in the avoidance zone in stressed animals than handled animals, but no effect of AAV‐pd‐GluA1. (e) There was a trend for a higher frequency of entries into the interaction zone in stressed rats compared to handled rats, but no effect of AAV‐pd‐GluA1. (f) There was a trend for higher cumulative duration of time spent in the interaction zone in stressed animals than handled animals, but no effect of AAV‐pd‐GluA1

A social interaction test was performed three days after the last stress or handling procedure in rats with or without AAV‐pd‐GluA1 viral infusions (Figure 5a–d). A two‐way ANOVA revealed a main effect of stress on number of entries into the avoidance zone (*n* = 55, *F*_1,51_ = 22.03, *p* < .0001), with stressed animals entering the avoidance zone significantly more times than did handled animals (*p* < .05). There was, however, no effect of AAV‐pd‐GluA1 (*F*_1,51_ = 2.443, *p* = .1242), or interaction between factors (*F*_1,51_ = 0.1182, *p* = .7552; Figure 5c,d). A two‐way ANOVA also revealed that handled rats trended to enter the social interaction zone more times than did stressed rats (*p* > .05), and there was no effect of AAV‐pd‐GluA1 (*p* > .05). In addition,—a two‐way ANOVA revealed a main effect of stress on cumulative time spent in interaction zone (s) (*F*_1,51_ = 13.91, *p* = .0005), with handled animals spending significantly more time in the interaction zone than did stressed animals (*p* = .0143). There was, however, no effect of AAV‐pd‐GluA1 (*F*_1,51_ = 0.2932, *p* = .5905) or interaction between the two factors (*F*_1,51_ = 0.2131, *p* = .6463; Figure 5e,f),

### 3.3 Experiment 3.

3.3

#### 3.3.1 Verification of GluA1‐wt overexpression in VTA DA neurons using fluorescent immunohistochemistry

**Figure 6:**
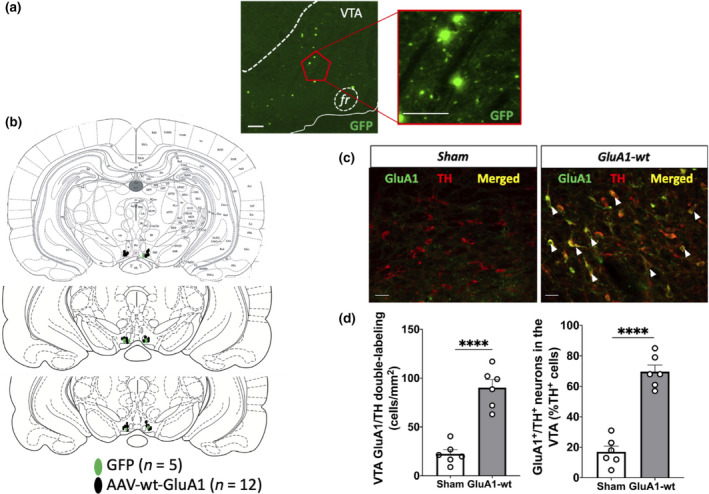
Verification of viral infusion sites: bilateral AAV‐wt‐GluA1 infusions into the VTA increases GluA1 expression in VTA DA neurons. (a) Representative fluorescent images showing GFP‐labelled cells in the VTA of a control rat at 5× objective magnification (left; scale bar: 100 µm) and 20× objective magnification (right; scale bar: 100 µm). (b) Schematic that depicts all viral infusion sites between −5.0 mm and −5.3 mm from bregma in control (green; *n* = 5) and experimental (black; *n* = 12) rats. (c) Because GluA1 antibodies recognize both active and inactive GluA1 AMPARs, representative fluorescent images show higher GluA1/TH double‐labelling in rats with cre‐dependent AAV‐wt‐GluA1 infusions (right) compared to sham (left)TH‐Cre rats handled (left) (bar = 50 µm; white arrow: GluA1/TH double‐labelled cell). (d) GluA1/TH double‐labelling in VTA is significantly higher in animals with AAV‐wt‐GluA1 infusions compared to sham rats (**** *p* < .0001)

Fluorescent immunohistochemistry revealed that AAV‐wt‐GluA1 infusions increased GluA1 expression in VTA DA neurons (Figure 6c,d). An **unpaired *t* test** was performed to compare the mean number of GluA1‐expressing DA neurons in sham compared to GluA1‐wt‐expressing handled rats. Significantly more GluA1/TH double‐labelled cells were present in the rostral VTA in rats expressing GluA1‐wt handled rats compared to sham‐handled rats (*n* = 12; *t*(10) = 7.323, *p* < .0001; Figure 6d). In addition, an **unpaired *t* test** was performed to compare the percentage of GluA1/TH double‐labelled neurons out of the entire TH neuronal population. There was a significantly higher percentage of GluA1/TH double‐labelled neurons in GluA1‐wt‐expressing handled rats compared to sham‐handled rats (*t*(10) = 9.208, *p* < .0001; Figure 6d). Thus, bilateral AAV‐wt‐GluA1 infusions were accurately performed in the VTA (Figure 6) and viral infusions increased GluA1 expression in DA neurons.

#### 3.3.2 Wild‐type GluA1 overexpression in VTA DA neurons mimics the effects of intermittent social defeat stress on AMPH cross‐sensitization

**Figure 7:**
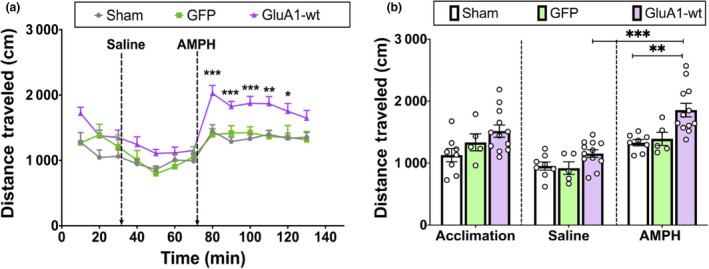
Functional GluA1 overexpression in VTA dopamine neurons by AAV‐wt‐GluA1 induces heightened locomotor activity in response to AMPH administration, even in the absence of intermittent social defeat stress. (a) AAV‐wt‐GluA1‐infused rats travelled a significantly greater distance than control groups 10–50 min after AMPH injection (***p < .0005, **p < .005, *p < .05). (b) There was no difference in locomotor activity during baseline or after saline injections (*p* > .05)

Comparing the effect of AMPH challenge in handled rats with or without prior Cre‐dependent AAV‐wt‐GluA1 infusion revealed a significant main effect of experimental group (*n* = 27, *F*_2,308_ = 47.04, *p* < .0001; Figure 7a) and time point on locomotor activity *(F_12_*_,308_ = 11.34, *p* < .0001), but no interaction between the two factors (*F*_24,308_ = 0.8694, *p* = .6446). Specifically, rats with GluA1 overexpression had significantly more locomotor activity than control/handled sham and handled/GFP rats at 10 (*p* = .0003), 20 (*p* = .0009), 30 (*p* = .0008), 40 (*p* = .0044) and 50 min (*p* = .0145) after AMPH challenge. There was no difference in locomotor activity over time in the control sham and handled/GFP groups (*p* > .05). Additionally, comparison of the average distance travelled during acclimation, after saline treatment and after AMPH challenge in the three different experimental groups by two‐way ANOVA revealed a significant main effect of experimental group (*F*_2,22_ = 6.295, *p* = .0069) and time point (*F*_2,44_ = 52.78, *p* < .0001; Figure 7b), as well as an interaction between the two factors (*F*_4,44_ = 3.182, *p* = .0222).

## 4 DISCUSSION

In this study, we assessed the role of AMPAR GluA1 subunits expressed by VTA DA neurons on social stress‐induced psychostimulant cross‐sensitization. The present results demonstrate that virus‐mediated functional inactivation of GluA1 expression in VTA DA neurons prevents social stress‐induced AMPH cross‐sensitization, and overexpression of wild‐type AMPAR GluA1 in VTA DA neurons augments locomotor response to AMPH, mimicking the effect of social stress. These results indicate that GluA1, specifically in VTA DA neurons, is both necessary and sufficient to induce stress‐induced sensitization to AMPH in male rats. Furthermore, manipulation of VTA GluA1 did not alter social avoidance behaviour, suggesting that the effect of GluA1 in VTA DA neurons is behaviourally specific.

### 4.1 Intermittent social defeat stress induces GluA1 expression in VTA DA neurons

The VTA is heterogeneous in cellular composition and is comprised of 60%–65% dopaminergic, approximately 35% GABAergic, and a small percentage of glutamatergic neurons (Nair‐Roberts et al., 2008). However, this heterogeneity is further complicated by the fact that VTA DA neurons may co‐release glutamate (Stuber, Hnasko, Britt, Edwards, & Bonci, 2010; Zhang et al., 2015) or GABA (Stamatakis et al., 2013; Tritsch, Ding, & Sabatini, 2012). In rodents, psychostimulant administration increases glutamate transmission in portions of the mesocorticolimbic pathway involved in behavioural sensitization to psychostimulants, including the NAc and VTA (Xue et al., 1996; Reid et al., 1997; Del Arco et al., 1999; Wolf & Xue, 1999). VTA glutamate input mostly arises from prefrontal cortical regions to modulate VTA DA neurons. Repeated stress has been shown to increase neuronal activity of VTA DA neurons which project to the NAc (Tidey & Miczek, 1997), as well as enhance glutamatergic synaptic plasticity through NMDA receptors (NMDARs) in the VTA (Stelly, Pomrenze, Cook, & Morikawa, 2016). The observed elevation of VTA GluA1 expression following exposure to social stress is consistent with previous studies, which revealed increased GluA1 protein expression in the VTA of animals subjected to chronic restraint, unpredictable stress and repeated social defeat stress (Covington et al., 2008; Fitzgerald, Ortiz, Hamedani, & Nestler, 1996; Wang et al., 2014). Glutamatergic synapses on VTA DA neurons undergo LTP that is enhanced by acute stress (Graziane, Polter, Briand, Pierce, & Kauer, 2013; Luscher & Malenka, 2011; Niehaus, Murali, & Kauer, 2010), while social stress augments DA release in the NAc (Tidey & Miczek, 1996; Miczek et al, 2011). In addition, cocaine administration induces LTP at excitatory synapses on VTA DA neurons (Argilli, Sibley, Malenka, England, & Bonci, 2008; Ungless, Whistler, Malenka, & Bonci, 2001) Thus, the enhancement of GluA1 expression in VTA DA neurons likely increases dopaminergic activity and plays a critical role in stress‐induced sensitization to AMPH. We did not observe GluA1 labelling in all TH + neurons, which might suggest that GluA1 expression also occurs in TH‐GABA or TH‐glutamate co‐expressing neurons because there are populations of VTA neurons that co‐express TH and GAD or VGlut2 (Morales & Margolis, 2017). In addition, it is possible that GluA1 may also be localized in VTA GABA cells, which could indirectly influence dopaminergic transmission. Further studies must be conducted to analyse GABA/GluA1 labelling density to confirm that GluA1 is selectively localized in VTA DA neurons as a result of intermittent social defeat stress.

### 4.2 Functional inactivation of GluA1 AMPARs in VTA DA neurons attenuates social stress‐induced cross‐sensitization, but has no effect on social avoidance behaviour

Stressed rats exhibited significantly greater locomotor activity following AMPH challenge compared to handled rats (Figure 2a), confirming the results of prior studies (Covington & Miczek, 2001; Nikulina et al., 2004). By contrast, viral overexpression of pd‐GluA1 in VTA DA neurons significantly attenuated stress‐induced AMPH sensitization. GluA1‐homomeric AMPARs are Ca^2+^‐permeable and are activated when glutamate occupies at least two of its binding sites (Wolf & Tseng, 2012). Phosphorylation of GluA1 at Ser^831^ by CaMKII and PKC during LTP facilitates delivery of GluA1‐containing AMPARs to the synapse (Hayashi et al., 2000) and increases single‐channel conductance (Derkach, Barria, & Soderling, 1999). It has been suggested that elevated intracellular Ca^2+^ signalling mediated by an increase of GluA1 in the VTA might be an early trigger for drug sensitization (Carlezon & Nestler, 2002). The induction of psychostimulant sensitization is blocked by systemic AMPAR antagonists (Li et al., 1997; Zhang et al., 1997) and repeated cocaine treatment increases GluA1 expression in the VTA one day later (Churchill, Swanson, Urbina, & Kalivas, 1999; Di Chiara & Imperato, 1988). Although drug sensitization is *initiated* in the VTA by enhanced glutamatergic transmission (Cador, Bjijou, Cailhol, & Stinus, 1999), overexpression of wt‐GluA1 in the NAc diminishes cocaine sensitization, while functional inactivation of NAc GluA1 enhances sensitization (Bachtell et al., 2008). Thus, these effects of GluA1 in the NAc are opposite to those in the VTA as demonstrated herein and previously (Churchill et al., 1999). Our data suggest that social stress‐induced GluA1 expression specifically in VTA DA neurons plays a critical role in the induction of stress‐induced cross‐sensitization to AMPH.

Furthermore, stress induces glucocorticoid release, which acts on mesolimbic DA neurons to exacerbate the locomotor effects of addictive drugs (Kalivas, 2009; Miczek, Yap, & Covington, 2008). It is likely this, along with the glutamatergic potentiation of VTA DA neurons, drives stress‐induced sensitization to AMPH. It is important to note that GluA1 AMPARs in the VTA are not the only glutamatergic mediators of social stress‐induced sensitization to psychostimulants. Metabotropic glutamate receptors located in the VTA have been shown to modulate cocaine‐induced synaptic plasticity (Loweth, Tseng, & Wolf, 2013; Mameli et al., 2009), and NMDA receptors in the VTA are necessary for stress‐induced behavioural sensitization in mice (Yap, Covington, Gale, Datta, & Miczek, 2005). Additionally, VTA NMDARs have been shown to mediate cocaine‐induced VTA GluA1 expression (Guzman et al., 2018) through NMDAR‐brain derived neurotrophic factor (BDNF)‐TrkB signalling, which mediates LTP of excitatory AMPAR input to VTA DA neurons after systemic cocaine injections (Pu, Liu, & Poo, 2006). In addition, increased BDNF expression has been implicated as a long‐term mediator of social stress‐induced AMPH cross‐sensitization (Nikulina et al., 2012) and knockdown of BDNF receptor TrkB in the mesolimbic pathway prevents GluA1 expression in the VTA (Wang et al., 2014). It is likely that corticolimbic glutamate signalling onto GluA1 AMPARs in VTA DA neurons works in parallel with these other mesolimbic mechanisms to induce the stress cross‐sensitization.

The present results show that functional inactivation of GluA1 in VTA DA neurons prevents stress‐induced sensitization to AMPH, but does not alter social avoidance behaviour. While the VTA does regulate stress‐induced social avoidance behaviour, this could involve different mediators of VTA function, such as mu‐opioid receptors (Johnston, Herschel, Lasek, Hammer, & Nikulina, 2015) or BDNF (Berton et al., 2006; Fanous et al., 2011; Krishnan et al., 2007), whose knockdown in the VTA prevents stress‐induced social avoidance behaviour. In addition, it was recently shown that the function of VTA dopamine neurons depends on distinct inputs and targets (Edwards et al., 2017; Lammel et al., 2012) that could reflect a diverse regulation of social stress‐induced avoidance behaviour and AMPH sensitization.

### 4.3 Overexpression of wild‐type GluA1 AMPARs in VTA DA neurons mimics the effects of stress on AMPH sensitization

Handled rats with VTA GluA1 overexpression exhibited significantly greater locomotor activity after a low dose AMPH challenge compared to handled rats with non‐manipulated VTA GluA1. These results are consistent with the idea that overexpression of GluA1 in VTA DA neurons is sufficient to mimic the effects of social stress on sensitization to psychostimulants. Importantly, there is a sharp increase of locomotor activity immediately after low dose AMPH challenge (Figure 6), which is a more pronounced response than we see in non‐manipulated stressed animals. Similarly, wild‐type GluA1 overexpression in regions of the VTA rich in DA neurons increases cocaine self‐administration (Choi et al., 2011). When GluA1 homomeric, Ca^2+^‐permeable AMPARs are overexpressed in VTA DA cells, there is an enhanced substrate to which glutamate can bind, thereby increasing intracellular Ca^2+^ signalling via CaMKII. CaMKII is heavily implicated in modulating long‐term‐potentiation and is involved in molecular mechanisms of addiction in the mesolimbic pathway; blocking CaMKII in the VTA has been shown to inhibit the acquisition of cocaine conditioned place preference and cocaine‐evoked synaptic plasticity in the NAc (Kourrich, Rothwell, Klug, & Thomas, 2007; Liu et al., 2014). Additionally, CaMKII activity in the NAc is essential for reinstatement of cocaine‐seeking in self‐administration, increasing AMPAR phosphorylation at Ser^831^, which is also obtained through viral overexpression of CaMKII in the NAc (Anderson et al., 2008; Loweth et al., 2010). It could be that overexpression of GluA1 in VTA DA cells drives the activation of intracellular CaMKII‐dependent Ca^2+^ pathways, which induces potentiation of VTA‐NAc DA neurons. In support of this, studies have shown that potentiation onto hippocampal neurons is largely driven by a CaMKII‐mediated augmentation of GluA1 surface expression (Appleby et al., 2010); so not only does GluA1 AMPAR signalling induce heightened CaMKII activity, but CaMKII also drives further insertion of GluA1 into the cell membrane resulting in further synaptic potentiation. Our behavioural results are consistent with previous findings that indicate that GluA1 plays a role in the induction of drug sensitization, but presents novel findings regarding its involvement in social stress‐induced AMPH sensitization.

### 4.4 Concluding remarks

In summary, functional inactivation of GluA1 in VTA DA neurons prevents social stress‐induced cross‐sensitization to AMPH but has no effect on social avoidance behaviour. In rats, repeated social defeat stress enhances glutamatergic synaptic plasticity in the VTA (Stelly et al., 2016), and GluA1 AMPARs play an essential role in the induction of drug sensitization and self‐administration (Carlezon & Nestler, 2002; McCutcheon, Wang, Tseng, Wolf, & Marinelli, 2011). Additionally, higher GluA1 expression occurs concomitantly with intermittent social stress‐induced AMPH sensitization (Wang et al., 2014). The present results show that GluA1 AMPARs in VTA dopaminergic neurons play an essential role in the induction of social stress‐induced psychostimulant sensitization.

Much of the work that has been conducted on the role of GluA1 in drug addiction has been focused on the NAc, which has reciprocal projections to the VTA. As our bilateral VTA GluA1 inactivation in the present study could have blocked the stress‐induced increase of dopaminergic tone, these bidirectional viral manipulations might alter stress‐induced changes in the NAc. Further studies are necessary to identify whether VTA GluA1 inactivation or overexpression causes neuronal changes in the NAc (e.g., prolonged ΔFosB expression). Additionally, due to the heterogenous nature of the VTA, GluA1 viral manipulations in other VTA cell type are necessary to determine whether GluA1 effects are specific to DA neurons.

In conclusion, our results present the novel finding that GluA1, specifically in VTA DA neurons, plays a critical role in the expression of social stress‐induced AMPH sensitization, thereby providing a potential target for pharmacotherapeutic intervention for drug abuse susceptibility.

## CONFLICT OF INTEREST

There is no conflict of interest to disclose.

## AUTHOR CONTRIBUTIONS

Megan Rudolph performed the aforementioned experiments, performed statistical analyses and wrote the manuscript. Rachael Neve designed the viral constructs that were used in these experiments. Ella Nikulina designed the study, provided assistance and experiment oversight, assisted in statistical analyses and data interpretation and edited the manuscript. Ronald Hammer was involved in study design, as well as experiment oversight, and manuscript editing.

## Data Availability

We will make our original data available as necessary. The peer review history for this article is available at https://publons.com/publon/10.1111/ejn.14884
